# The peritoneal cestode *Taenia crassiceps* restructures gut bacterial communities in the mouse host: identification of potential resistance-associated bacteria

**DOI:** 10.1007/s00436-025-08574-1

**Published:** 2025-10-22

**Authors:** Diego Mateos-Arenas, Miguel Ruiz-de la Cruz, Héctor Martínez-Gregorio, Marisol I. González, Felipe Vaca-Paniagua, Clara E. Díaz-Velásquez, Bertus Eksteen, Danielle Vannan, Luis I. Terrazas, José L. Reyes

**Affiliations:** 1 Laboratorio de Inmunología Experimental y Regulación de La Inflamación Hepato-Intestinal, UBIMED, FES Iztacala, UNAM, Estado de México, Tlalnepantla de Baz, Mexico; 2 Laboratorio de Genómica Integrativa del Cáncer y Enfermedades Infecciosas, UBIMED, FES Iztacala, UNAM, Estado de México, Tlalnepantla de Baz, Mexico; 3 Aspen Woods Clinic, Calgary, Alberta Canada; 4 Boston Scientific Corporation, Urology Division, 200 Boston Scientific Way, Marlborough, MA USA; 5 Laboratorio de Inmunoparasitología, UBIMED, FES Iztacala, UNAM, Estado de México, Tlalnepantla de Baz, Mexico

**Keywords:** Microbiota, *Dubosiella*, NLRP3, *Taenia crassiceps*

## Abstract

Helminthic parasites cause chronic infections due to their ability to generate immune regulatory networks in their hosts; however, an additional component in this interplay is the microbiota. In this regard, new insights have emerged from intestinal helminthic infections, but whether extraintestinal worms are able to alter intestinal microbiota is unknown. Here, we explored microbiota changes occurring during experimental infection with the peritoneal, non-migrating cestode *Taenia crassiceps*. Our sequencing approach allowed for the identification of 56 families and 119 genera in the fecal samples. We compared the microbiota composition between wild-type (WT) and NLRP3-deficient mice (NLRP3^−/−^), since we recently reported that the lack of NLRP3 dramatically enhanced mouse resistance against *T. crassiceps*. Prior to *T. crassiceps* infection, NLRP3^−/−^ mice harbored distinct microbial communities when compared to WT animals. The lack of NLRP3 resulted in enrichment of *Akkermansia* and *Lachnospira*, while reducing *Clostridium*, *Dubosiella*, and *Bifidobacterium*. Strikingly, *Lactobacillus* presented a comparable abundance in uninfected WT and NLRP3^−/−^ mice. Upon infection, a progressive increase in *Akkermansia* and a transitory expansion of *Lachnospiraceae* were observed in WT mice but not in NLRP3^−/−^ mice. A remarkable finding was that *T. crassiceps* infection inhibited *Dubosiella,* whereas the groups with enhanced resistance to *T. crassiceps* (NLRP3^−/−^ mice and WT individuals co-housed with NLRP3^−/−^ mice) presented a greater abundance of *Dubosiella,* indicating that resistance against *T. crassiceps* might be coordinated by this bacterium. Our study reveals that intestinal microbiota can also be modulated through a peritoneal-gut axis, in which the players (antigens and immune cells) remain to be identified. In addition to resistance, microbiota changes could be involved in the *T. crassiceps*-evoked anti-inflammatory and anti-oncogenic effects.

**Supplementary Information:**

The online version contains supplementary material available at 10.1007/s00436-025-08574-1.

## Introduction

Helminthic parasites can chronically infect their hosts by eliciting immune-regulatory networks consisting of cell populations with altered features, both favoring parasite growth while attenuating host damage (Zakeri et al. 2018). Although manipulating immune cell populations is key for helminthic parasites, other host-related components, such as microbiota, are also targeted by worms (Gause and Maizels 2016). Salient evidence shows a highly specialized interplay between helminths and microbiota in several gastrointestinal (GI) compartments. It has been reported that there are changes in microbiota in the ileum of mice infected with *Heligmosomoides polygyrus* (Walk et al. 2010) and in response to the migrating larvae of *Nippostrongylus brasiliensis*, where fundamental components of the Th2-type response, such as IL-13 and the transcription factor STAT6, were necessary to inhibit filamentous bacteria growth (Fricke et al. 2015). Furthermore, chronic infection with *Trichuris muris* in the mouse colon alters the local microbiota. Interestingly, reducing bacterial load by means of antibiotic exposure resulted in lower egg hatching, showing that co-evolving with the host’s bacteria is beneficial for *T. muris* (Hayes et al. 2010). Moreover, additional aspects of this intricate relationship were uncovered, given that host-derived bacteria are internalized by *T. muris* (White et al. 2018). Of note, this helminth-bacteria crosstalk has been demonstrated to occur in diverse vertebrate hosts, such as pigs and livestock (Williams et al. 2021). Also, human studies show that helminthic parasites modulate the abundance of bacterial communities in cohorts of children (Guernier et al. 2017; Stracke et al. 2021). Therefore, salient evidence shows significant changes in microbiota during intestinal worm infections, apparently with diverse purposes and consequences.

Nevertheless, several helminthic parasites reside in diverse extra-intestinal anatomical niches in their hosts, for instance, the filarial nematode *Litomosoides sigmodontis* in the pleural cavity (Finlay et al. 2023) and the cestode *Taenia crassiceps* in the peritoneal cavity (Peon et al. 2016). However, whether extra-intestinal worm parasites elicit changes in the intestinal microbiota has been poorly explored.

We recently reported that the lack of NLRP3 resulted in enhanced resistance against the peritoneal cestode *T. crassiceps*, and co-housing experiments suggested that intestinal microbiota may be involved (Flores-Sotelo et al. 2024). Harnessing this model, we aimed to assess microbiota changes in mice infected with *T. crassiceps* and to compare them with resistant NLRP3^−/−^ mice. Here, we report the resulting microbiota differences in mice lacking NLRP3 under steady conditions, as well as the redistribution of bacterial communities after peritoneal infection with *T. crassiceps*.

## Methods

### Fecal sample collection

Due to the high susceptibility displayed by female mice in the context of *Taenia crassiceps* infection as compared to male mice, 8-week-old female wild-type (WT) and NLRP3-deficient (NLRP3^−/−^) mice (mouse colony donated by Dr. Daniel Muruve, University of Calgary, AB, Canada) were maintained in germ-free facilities with controlled temperature (20 °C) and light cycles (12 h/12 h) according to the federal guidelines for experimental animal care (NOM-062-ZOO-1999), and the use of these organisms was approved by our faculty’s Bioethics committee (CE/FESI/042022/1513). These mice were infected with 20 metacestodes harvested from 8-week-infected BALB/c female mice and followed for eight weeks. To collect fecal samples, animal cages were transferred into aseptic conditions, and thereafter, fecal pellets from animals were immediately collected in sterile tubes (Eppendorf, Germany) and snap-frozen in liquid nitrogen. These fecal samples were collected from animals employed in a previous study where enhanced resistance in mice lacking NLRP3 was evident (Flores-Sotelo et al. 2024).

### Assessment of intestinal microbiota

DNA was obtained from 10 mg of each fecal sample using the DNeasy blood and tissue kit (Qiagen, Germany) following the manufacturer’s instructions. Briefly, upon sample incubation with lysozyme-containing lysis buffer (20 min at 37 °C), proteinase K was added for an additional incubation (30 min at 56 °C), and remaining RNA was removed by adding RNase to the samples. Finally, samples were eluted in DNase- and RNase-free water (100 µl) to quantify DNA concentration in each sample by fluorometric means (Quantus, Promega, USA). Once DNA concentrations were adjusted, the integrity of samples was verified on 1.2% agarose gel stained with ethidium bromide (Sigma-Aldrich, USA). Next, using 50 ng DNA template from each sample, the amplification of DNA fragments from the V3–V4 subunits of the 16S ribosomal RNA gene was carried out through end-point PCR. The universal adapter-containing sequences were forward: TCGTCGGCAGCGTCAGATGTGTATAAGAGACAGCCTACGGGNGGCWGCAG and reverse: GTCTCGTGGGCTCGGAGATGTGTATAAGAGACAGGACTACHVGGGTATCTAATCC (Illumina, USA). A specific 550 bp DNA fragment was observed in all samples, as shown in Supplementary Fig. 1. DNA libraries were purified with 0.8% AMpure pearls, and molecular labeling was carried out with P5 and P7 barcodes (Illumina, USA). Finally, mass sequencing was conducted on a MiSeq platform with a 2 × 250 paired-end format, yielding a 100,000 × theoretical depth (Illumina, USA). Mass sequencing data were uploaded and are available in the NCBI database, SRA code PRJNA1268537. Bioinformatics analysis was conducted using QIIME2 software, and abundance graphs (only bacterial genera with ≥ 1% abundance are shown) were created using the RStudio software (v4.5.0). We based our discussion on changes found in terms of the abundance of bacterial genera in the fecal samples.

## Results and discussion

It has been shown that parasitic intestinal worms have co-evolved into a close relationship with surrounding bacteria once they become established in the GI tract. This interplay includes the acquisition of microbial communities by these parasites and suggests a bidirectional benefit (Hayes et al. 2010). Whether worm parasites inhabiting distant tissues are able to restructure intestinal microbiota upon infection has not been addressed. However, Doonan et al. reported that subcutaneous inoculation of the antigen ES-62 from the nematode *Acanthocheilonema viteae* reverted the intestinal microbiota changes caused by collagen-induced experimental arthritis in DBA/1 mice, suggesting far-reaching effects of helminth-derived secreted antigens on intestinal microbiota composition (Doonan et al. 2019).

**Fig. 1:**
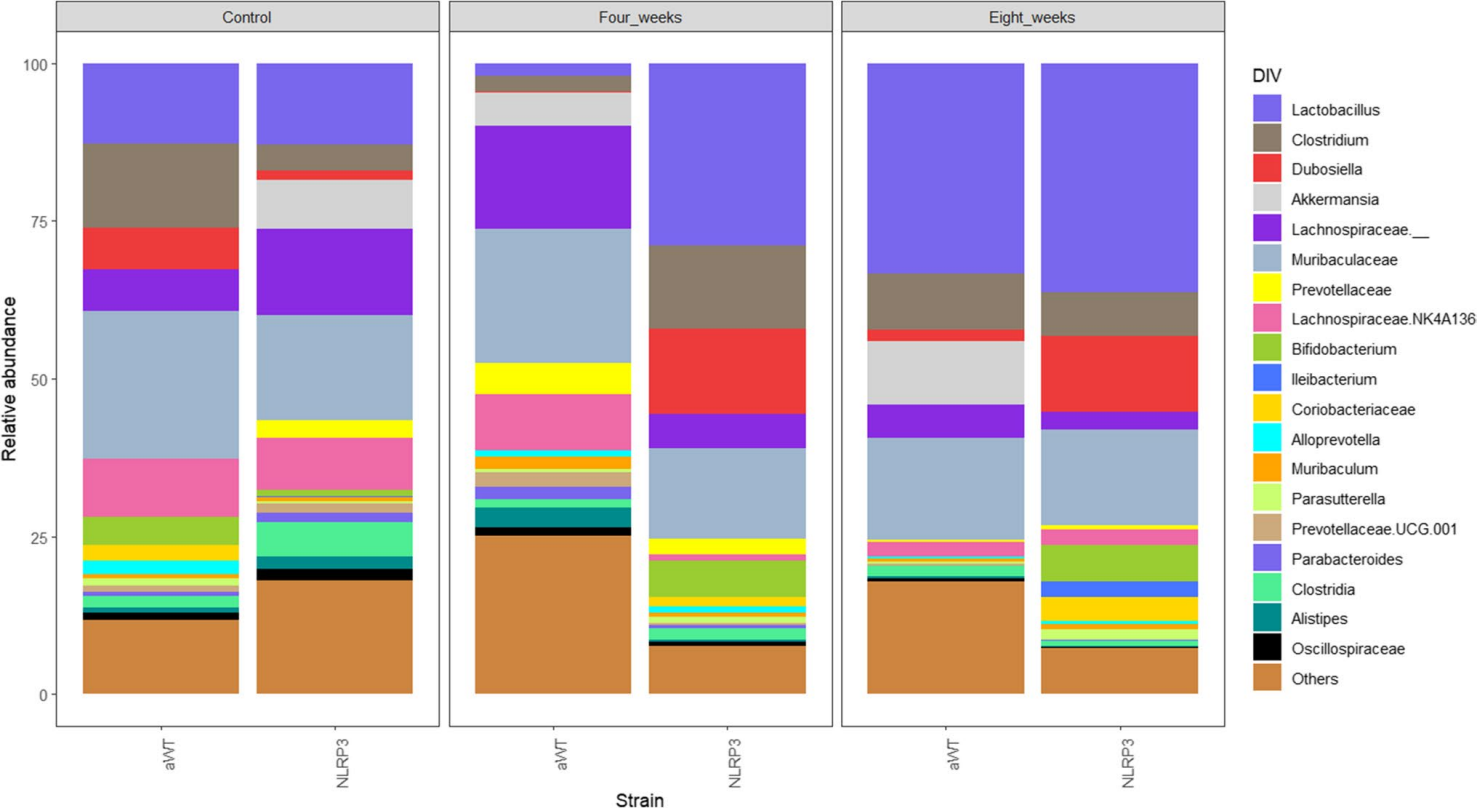
The peritoneal infection with *T. crassiceps* parasites alters the mouse native intestinal microbiota. The 16S rRNA subunit sequencing allowed the determination of the relative abundance of bacterial genera prior to infection as well as at 4 and 8 weeks post-infection in both WT and NLRP3^−/−^ mice, as shown

First, we compared the bacterial abundance between female WT and NLRP3^−/−^ mice prior to infection, and several differences were evident. The WT mouse colony, compared to sex- and age-matched NLRP3^−/−^ mice maintained under the same conditions, showed, on average, significantly increased abundance of the following bacteria: *Clostridium* (WT 13.3% vs. NLRP3^−/−^ 4.1%), *Dubosiella* (WT 6.6% vs. NLRP3^−/−^ 1.5%), *Bifidobacterium* (WT 4.3% vs. NLRP3^−/−^ 0.9%), and *Alloprevotella* (WT 2% vs. NLRP3^−/−^ 0.05%), as shown in Fig. 1. Conversely, NLRP3^−/−^ mice, when compared to WT littermates, showed enrichment in *Akkermansia (*WT 0% vs. NLRP3^−/−^ 7.75%*), Lachnospiraceae (*WT 6.62% vs. NLRP3^−/−^ 13.6%), and *Prevotellaceae* (WT 0.005% vs. NLRP3^−/−^ 2.8%) (see Fig. 1). Of note, the abundance of bacteria belonging to the *Lactobacillus* genus was comparable between WT and NLRP3^−/−^ mice under basal conditions (Fig. 1).

Previous reports have also conducted comparative microbiota analyses between WT and NLRP3^−/−^ mice from their respective facilities, and several differences are recognized. Contrary to our findings, Hirota et al. found increased *Clostridium* bacteria in female NLRP3^−/−^ mice as compared to their sex-matched WT counterparts (Hirota et al. 2011). Furthermore, Zhang et al. dissected the microbiota of both WT and NLRP3^−/−^ male mice (Zhang et al. 2019), and coincidentally reported that *Clostridium* and *Prevotella* were more abundant in NLRP3^−/−^ animals, as occurred in our experimental organisms. Interestingly, this microbiota profile was found to ameliorate behavioral disorders when fecal transplants were conducted (Zhang et al. 2019). Thus, several coincidences with other studies appeared in our microbiota characterization. The microbiota profile in our NLRP3^−/−^ mouse colony containing more *Akkermansia* is intriguing, however, it adds to several reports showing a negative feedback loop between NLRP3 and *Akkermansia* (Han et al. 2024).

In regard to microbial changes evoked by *T. crassiceps* infection, it was evident that a clear re-distribution of bacterial genera started at 4 weeks of infection. In WT mice, several bacterial genera showed increased abundance when compared between uninfected and 4 weeks-infected littermates as follows: *Akkermansia* (uninfected WT 0% vs. 4 weeks-infected WT 5.26%)*, Lachnospiraceae* (uninfected WT 6.6% vs. 4 weeks-infected WT 16.3%), and *Prevotellaceae* (uninfected WT 0.005% vs 4 weeks-infected WT 4.9%), while among others (i.e., *Lactobacillus* and *Clostridium*), *Dubosiella* reduced its abundance upon 4 weeks of infection, as shown in Fig. 1. In contrast, in NLRP3^−/−^ mice, whose resistance is superior and in which a significant reduction of parasite numbers is observed at 4 weeks of infection, a microbiota profile with abundant *Lactobacillus* (uninfected NLRP3^−/−^ 12.9% vs. 4-week-infected NLRP3^−/−^ 28.9%), *Clostridium* (uninfected NLRP3^−/−^ 4.1% vs. 4-week-infected NLRP3^−/−^ 13.1%), and *Dubosiella* (uninfected NLRP3^−/−^ 1.5% vs. 4-week-infected NLRP3^−/−^ 13.5%) prevailed. These data strongly indicate that suppressing bacteria such as *Lactobacillus*, *Clostridium*, and *Dubosiella* in the early stage may pave the way for a successful establishment of *T. crassiceps.* Interestingly, unlike *Dubosiella, Lactobacillus* and *Clostridium* bounced back later as the infection progressed (see Fig. 1). In line with this, the ileal presence of *Nippostrongylus brasiliensis* (11 days post-infection) significantly suppressed the abundance of *Clostridiaceae,* and a slight but significant increase in *Lactobacillus* was observed (Fricke et al. 2015). Furthermore, Walk et al. showed that the infection with *Heligmosomoides polygyrus* (2 weeks post-infection) resulted in significant changes in bacterial abundance in the ileum but not in the caecum. These authors observed an increase in *Lactobacillus* at 2 weeks of infection (Walk et al. 2010). Thus, intestinal nematodes cause *Lactobacillus* to increase in the acute stage of infection, whereas *T. crassiceps* limits the growth of these bacteria at 4 weeks of infection. However, this seems to be a transient phenomenon since these *Lactobacilli* exhibited greater abundance at 8 weeks of infection (see Fig. 1). Whether the abundance of *Lactobacillus* in intestinal nematode infections fluctuates over longer time periods or during secondary challenges where memory responses act, or whether modulating *Lactobacillus* is an antigen-specific effect, remains to be addressed.

At the chronic stage of *T. crassiceps* infection (8 weeks), the successful establishment of this cestode is caused by an intense Th2-type response (Rodriguez-Sosa et al. 2002a). Notably, components of the Th2-type response (e.g., STAT6) can also be a trigger for further changes in the microbiota composition (Fricke et al. 2015). The microbiota analysis in this study revealed a distinct microbiota landscape emerging in chronically infected WT animals as compared to 4-week-infected WT animals. The microbial signature present in chronically infected WT animals consisted of a further increased abundance of *Akkermansia* (4-week-infected WT 5.2% vs. 8-week-infected WT 10%), *Lactobacillus (*4-week-infected WT 10% vs. 8-week-infected WT 0%), and *Clostridium* (4-week-infected WT 2% vs. 8-week-infected WT 33.3%) (Fig. 1). On the other hand, the highly resistant NLRP3^−/−^ mice displayed only negligible differences in microbiota composition between 4 and 8 weeks of infection, wherein a sustained presence of *Dubosiella* (4-week-infected NLRP3^−/−^ 13.5% vs. 8-week-infected NLRP3^−/−^ 12.1%) and an undetectable abundance of *Akkermansia* was evident (4-week-infected NLRP3^−/−^ 0% vs. 8-week-infected NLRP3^−/−^ 0%) (Fig. 1). Therefore, the microbial signature present in chronically infected, susceptible WT mice (abundant *Akkermansia* and low *Dubosiella*) was completely inverted in resistant, time-matched NLRP3^−/−^ infected mice.

The finding that intestinal microbiota continues its re-distribution process from the acute (4 weeks) to chronic stage (8 weeks) of *T. crassiceps* infection is relevant since it has been demonstrated that *T. crassiceps* infection presents distinct immune responses in early stages (2 or 4 weeks) compared to chronic stages (8 and 12 weeks). Of note, the emergence of a strong Th2-type response and different macrophage subpopulations (Rodriguez-Sosa et al. 2002b), both of which are required to allow host colonization, can now be associated with time-dependent intestinal microbiota changes, which adds an additional component to the regulatory network generated by *T. crassiceps*. Although a direct effect of *T. crassiceps* on this bacterial balance is a possibility, we cannot rule out that these bacteria may also be regulating each other. The current challenge is to unveil the stepwise relationship among Th2 polarization, suppressive macrophage reprogramming, and microbiota re-distribution.

**Fig. 2:**
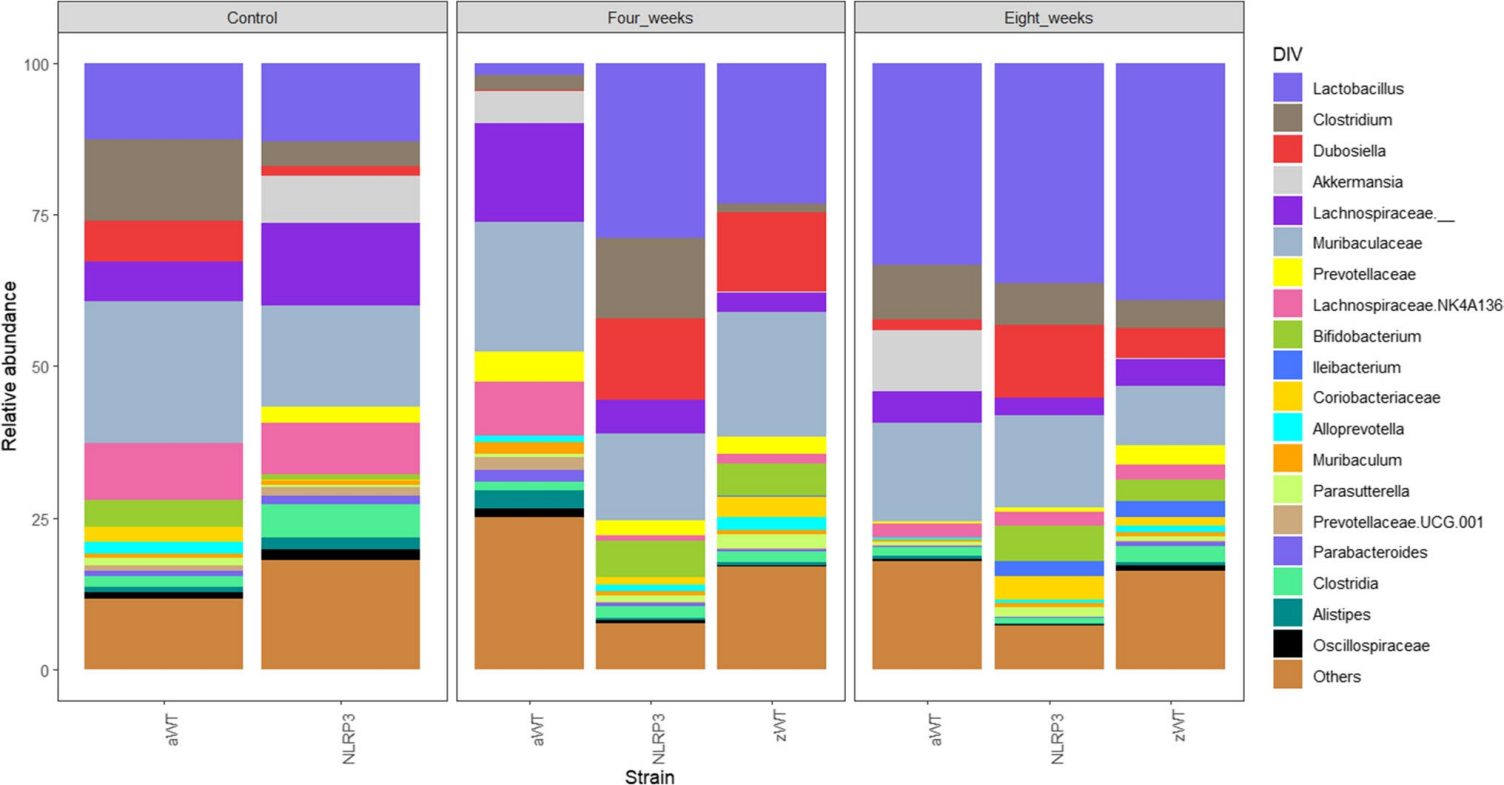
The persistence or acquisition of *Dubosiella *is associated with resistance to *T. crassiceps* infection. The relative abundance of *Dubosiella* was higher in naturally resistant NLRP3^−/−^ mice as well as in WT mice co-housed (WT CoH) with NLRP3^−/−^ mice, as compared to WT mice

Notably, we previously reported that the resistance against *T. crassiceps* may be transferred via intestinal microbiota exchange (Flores-Sotelo et al. 2024); this is indicative that one or more bacterial communities might be involved in this phenomenon. To identify any candidate potentially involved in promoting resistance, the microbiota profiles of WT, NLRP3^−/−^, and WT mice co-housed with NLRP3^−/−^ mice were analyzed and presented in Fig. 2. In such analysis, it was clear that bacteria from the genus *Dubosiella* prevailed only in resistant NLRP3^−/−^ mice, but more importantly, these bacteria were shown to be expanded in WT animals co-housed with NLRP3^−/−^ mice both at 4 weeks (WT 0.2% vs. NLRP3^−/−^ 13.5% vs. WT CoH 13.1%) of infection as well as 8 weeks (WT 1.8% vs. NLRP3^−/−^ 12.1% vs. WT CoH 4.9%). Intriguingly, a comparable pattern was observed with *Bifidobacterium* communities, although with lower abundance (Fig. 2). This strongly suggests that preserving or acquiring *Dubosiella* restrains *T. crassiceps* growth. Thus, these data clearly demonstrate a positive association between enhanced resistance and the presence of *Dubosiella,* posing this bacterium as a strong candidate for promoting limited colonization by T. *crassiceps*. The underlying mechanisms for this latter finding may be diverse; an intriguing possibility is that *Dubosiella* might be regulating the resistance against *T. crassiceps* through controlling sex hormones, as it has been shown to have a great effect on mouse sex hormone regulation in neurological disorders (Nguyen et al. 2025). Sex hormones are central in regulating susceptibility to *T. crassiceps* in the mouse model (Escobedo et al. 2004). Also, given the imperative need for macrophage reprogramming by *T. crassiceps*, these immune cells represent another potential target for *Dubosiella.* Further studies will uncover how *Dubosiella* influences the immune response during *T. crassiceps* infection. Likewise, whether *Bifidobacterium* plays any role along with *Dubosiella* as an additional resistance factor in this complex host-parasite interplay awaits future research.

In summary, it has been demonstrated that intestinal helminth-evoked microbiota changes are caused by several mechanisms such as intestinal barrier disruption (Schachter et al. 2020) and Th2 responses (Fricke et al. 2015). The peritoneal-dwelling cestode *T. crassiceps* can trigger time-dependent changes in several bacterial communities in the mouse host; these changes include bacterial communities being affected transiently and bacteria that expand progressively. Insight was gained in terms of resistance against *T. crassiceps*, and this may be extrapolated to the context of anti-inflammatory and anti-oncogenic features previously attributed to this parasite. Interestingly, the trigger for these microbial changes remains unknown, and we can only speculate on a likely bidirectional gut-peritoneum axis resulting from fluctuating immune responses, immune cell recirculation, and direct exposure to soluble antigens.

## Supplementary Information

Below is the link to the electronic supplementary material.

Supplementary file 1 (DOCX 603 KB)

## Data Availability

Mass sequencing data were uploaded and are available in NCBI database SRA code PRJNA1268537.
