# Supplementary material for: The peritoneal cestode Taenia crassiceps restructures gut bacterial communities in the mouse host: identification of potential resistance-associated bacteria
Source: Parasitol Res. 2025 Oct 22;124(10):116. doi: 10.1007/s00436-025-08574-1 (PMC12546395; doi:10.1007/s00436-025-08574-1)
Supplement: Supplementary file 1 — (DOCX 603 KB) [file 436_2025_8574_MOESM1_ESM.docx]

**
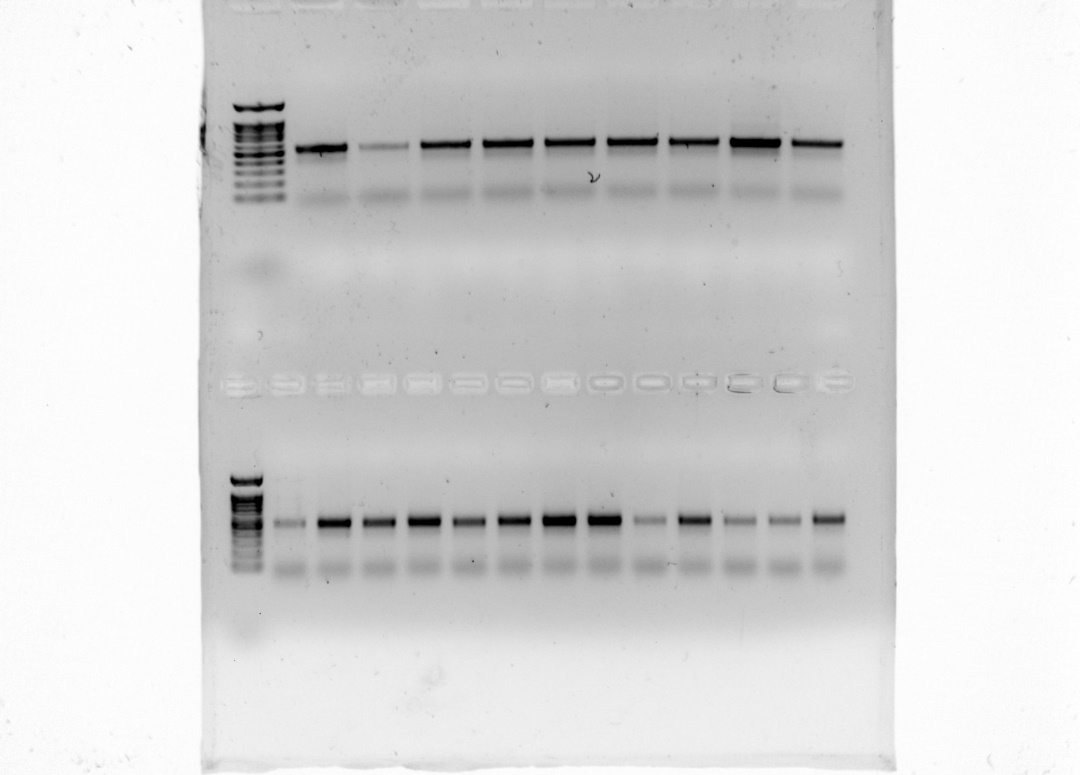
**

**
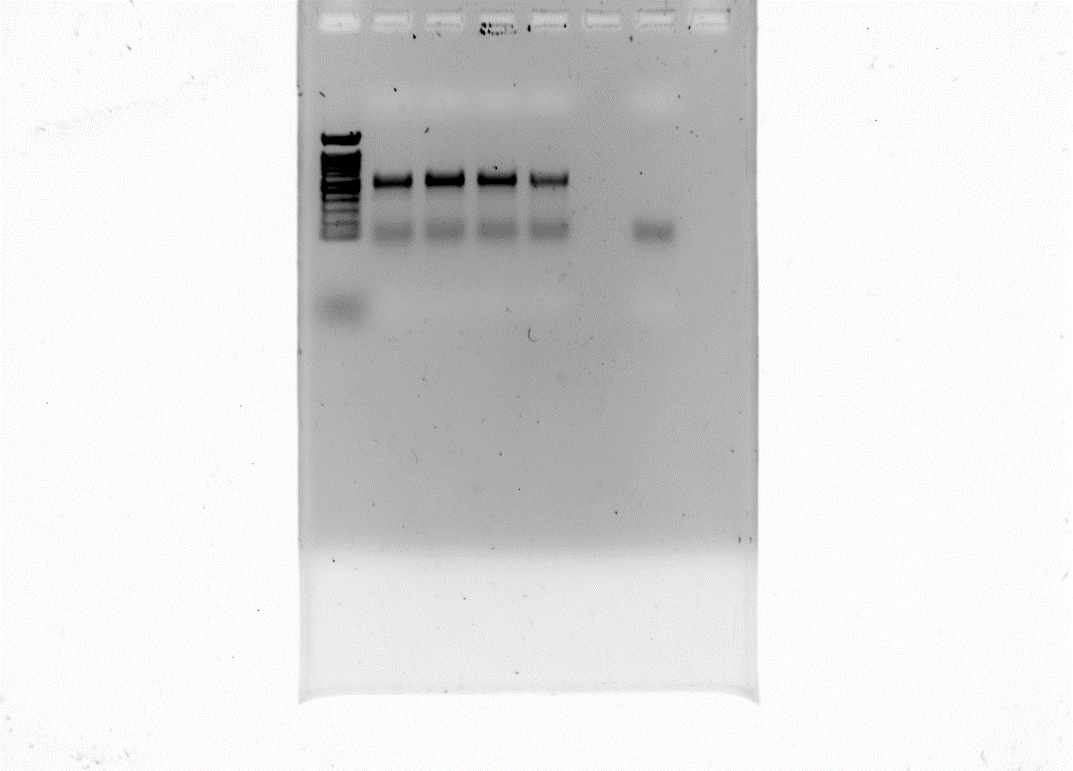
**

**Supplementary Figure 1.** PCR assay revealed a highly specific amplification of the expected 550 DNA fragment in all the 26 samples (deposited in NCBI database SRA code PRJNA1268537) and negative control (last lane on the righ) used in this study. Original agarose gels are shown.


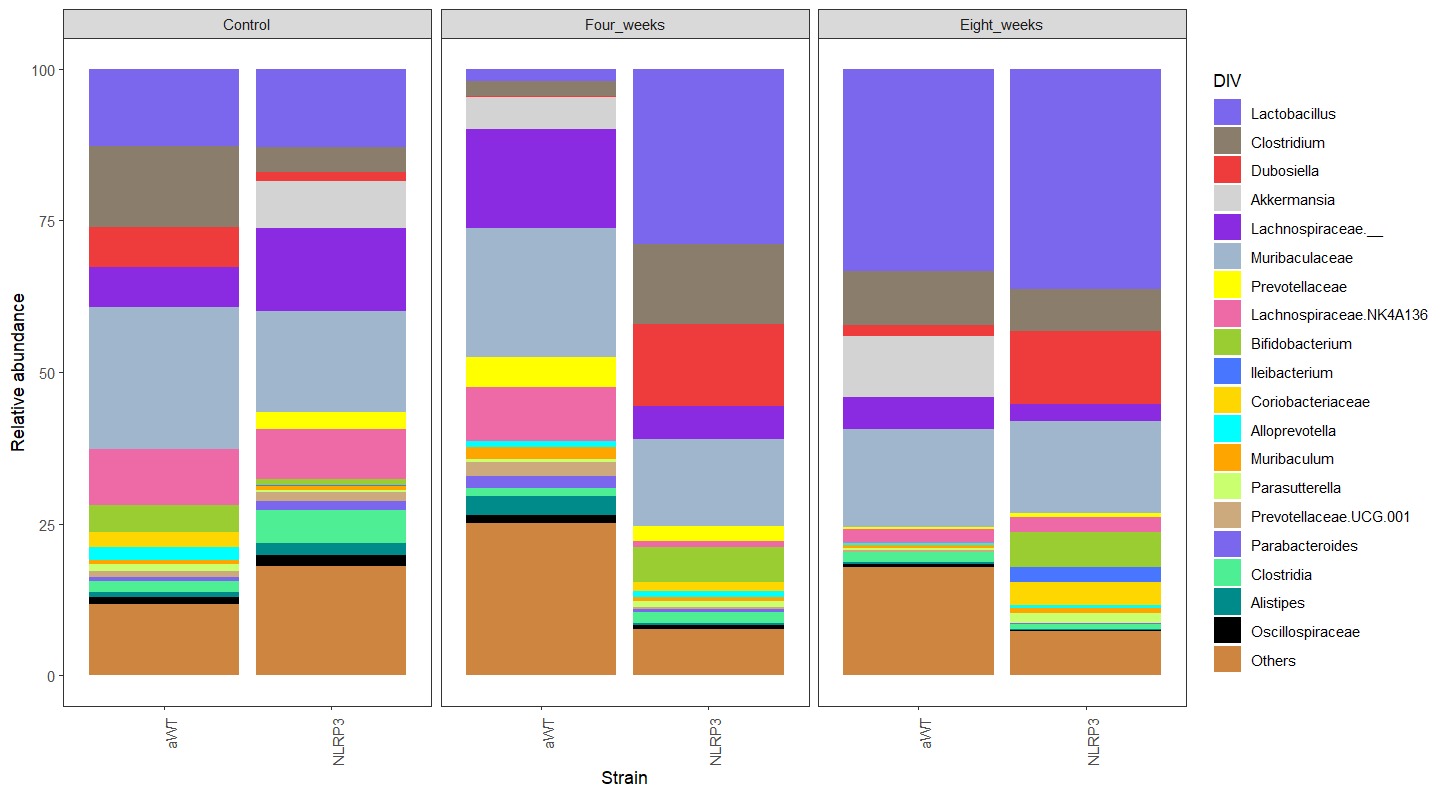


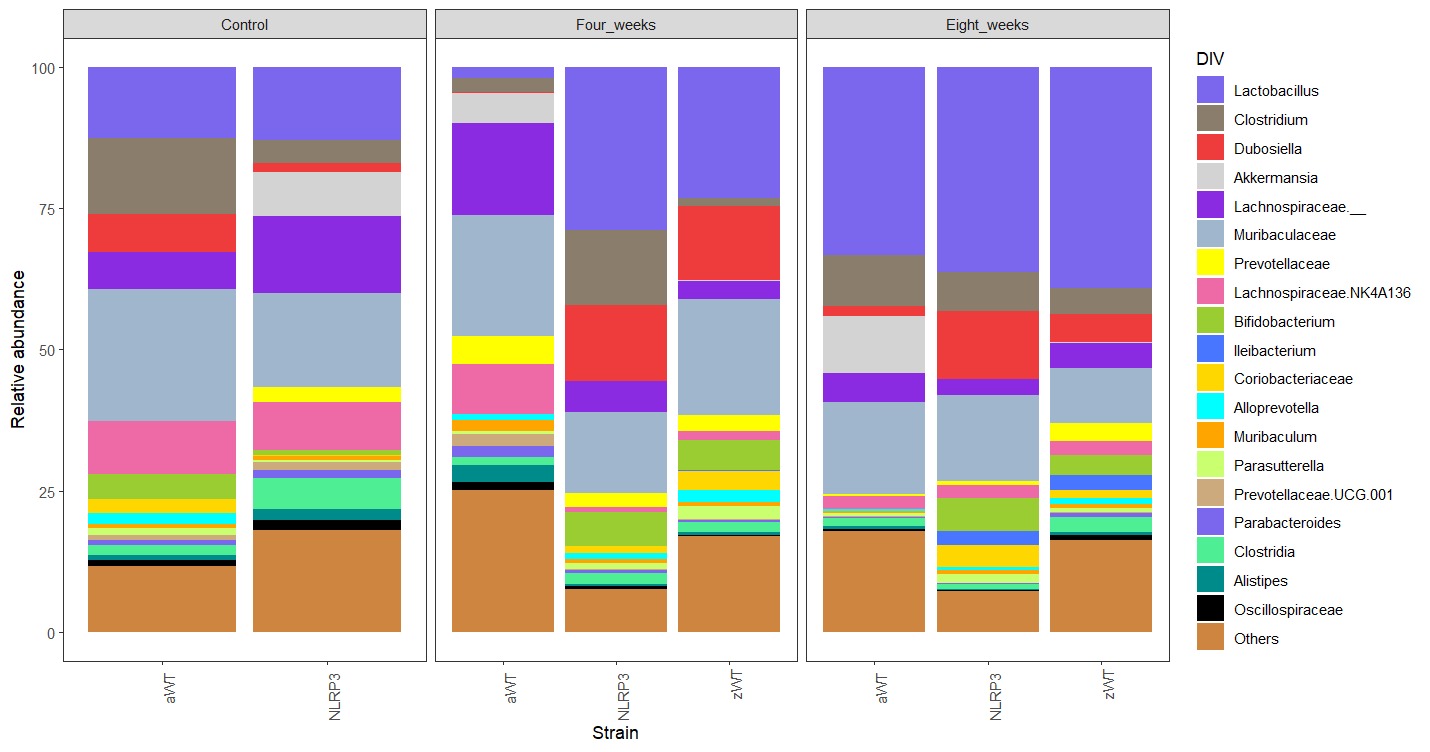


**Supplementary Figure 2.** Original relative abundance bar graph exported upon the bioinformatics analysis with RStudio software. The labels were added in order to be presented in the manuscript, the labels shown here are the ones automatically generated by the software. The corresponding samples are deposited in NCBI database SRA code PRJNA1268537.
